# Adverse childhood experiences negatively impact sustained attention in adulthood

**DOI:** 10.1007/s44192-025-00362-8

**Published:** 2026-01-14

**Authors:** Eicca Berentz, Christian Wienke, Tino Zaehle

**Affiliations:** 1 Institute of Medical Psychology, Medical Faculty, University Hospital Magdeburg, Otto-von-Guericke-University Magdeburg, Magdeburg, Germany; 2 Department of Neurology, Section Neuropsychology, Otto-von-Guericke University, Magdeburg, Germany; 3 Center for Behavioral Brain Sciences (CBBS), Magdeburg, Germany; 4 Research Group “Magdeburger Arbeitsgemeinschaft für Forschung unter Raumfahrt- und Schwerelosigkeitsbedingungen” (MARS), Magdeburg, Germany

**Keywords:** Sustained attention, Adverse childhood experiences, ACE, Gradual onset continuous performance task, GradCPT, Childhood trauma

## Abstract

**Background:**

Adverse childhood experiences (ACE) have been linked to reduced cognitive functioning in adulthood. This cross-sectional online study refines this picture by including emotional and physical domains of childhood trauma.

**Methods:**

A total of 262 individuals completed the German version of the Childhood Trauma Questionnaire (CTQ) to operationalize ACE. Sustained attention was measured using the gradual onset Continuous Performance Task (gradCPT). Multivariate linear regressions modeled sustained attention parameters (d’, RT, RTCoV, criterion) based on total CTQ score and emotional / physical subscores.

**Results:**

Higher CTQ Total Scores predicted lower discrimination performance (d′) and increased reaction time variability (RTCoV). No significant effects were found for the emotional and physical trauma subscores.

**Conclusion:**

These findings suggest lasting, negative ACE effects on sustained attention in adulthood. Additionally, the fully online design proved to be a reliable method for detecting subtle variations in sustained attention.

## Introduction

Traumatic experiences during childhood (described in the literature as adverse childhood experiences; ACE) are a major burden for affected people, even in adult life [3]. According to the definition by the World Health Organization (WHO), ACE are divided into abuse and neglect [25]. Abuse involves acts of caregivers that cause actual or potential harm and can further be divided into emotional, physical or sexual domains. Neglect involves the failure to provide a healthy environment for the child. It can be divided into an emotional and physical domain. Furthermore, a third domain of household dysfunction is often described, which includes numerous unfavorable constellations, such as substance abuse, death of a close relative or parental divorce [3]. Current research suggests that ACE influence neuronal development in childhood (see [15], for a review). Common impairments affect brain structure and functioning, particularly in the prefrontal cortex (PFC), which plays a central role in cognitive control, attention, and emotional regulation [7]. Neurobiological research has shown that ACE can disrupt white matter integrity and connectivity within large-scale brain networks, including the fronto-parietal network (FPN) and the default mode network [1, 9, 15]. It is, however, important to differentiate which type of abuse or neglect was involved. Physical and emotional ACE appear to have different effects on neuronal development [1, 14, 15]. In both dimensions, the PFC, including the anterior cingulate cortex (ACC), seems to be affected with a stronger impairment following emotional abuse and neglect. In particular, altered connectivity and integrity of the medial and dorsolateral PFC and the ACC were observed [1]. Emotional ACE are associated with abnormalities in fronto-limbic socioemotional networks which include the FPN, the default mode network (DMN), connections between these two networks and the visual network. On the other side, neglected individuals show reduced white matter integrity and connectivity in a heterogeneous set of brain areas. Impairments were shown in the parietal network, but also in the FPN and DMN. The visual network is less affected by neglect than by emotional trauma [1, 26].

While general neuroanatomical and -physiological consequences of ACE have been documented [14, 22], specific cognitive abilities associated with the PFC — such as sustained attention—have received less attention. Sustained attention refers to the ability to maintain consistent focus over prolonged periods and depends on the intact functioning of the PFC and associated networks [13, 19]. Only one study so far investigated effects of childhood traumatisation on sustained attention [24]. Here, ACE were operationalized as a general adversity score based on the presence and frequency of dysfunctional parental experiences (e.g., parental substance abuse, divorce, foster care). Sustained attention was assessed using the gradual onset continuous performance task (gradCPT, [2] a go/no-go task where complex visual stimuli constantly morph into each other without clear on- or offset. The authors observed significant negative effects of ACE on sustained attention, including reduced discriminatory ability, increased reaction time and greater reaction time variability as a function of the trauma score [24].

The aim of the current study is to expand on these results by differentiating between emotional and physical dimensions of ACE using the Childhood Trauma Questionnaire (CTQ) [12]. The CTQ allows for a more nuanced understanding of how emotional and physical ACE are uniquely associated with sustained attention performance. In addition to a CTQ total score, the emotional and physical subscale sum scores were examined individually, as recent studies suggest different effects of the ACE dimensions [15, 21]. As in Vogel et al. [24], sustained attention was measured using the gradCPT, an established marker of sustained attention that has been validated in a large-scale online study [5].

The present study makes several methodological and conceptual contributions to the literature on ACE and sustained attention. First, we distinguish between emotional and physical ACE domains, which allowed us to explore the association of different types of trauma with sustained attention and how the association may be differentially affected by trauma exposure. Second, instead of a composite adversity index, we use the well-validated CTQ to allow for psychometrically sound and standardized assessment of individual ACE subtypes. Third, the fully online administration of both the CTQ and the gradCPT datasets is methodologically novel allowing for large-scale and ecologically valid data collection across a range of devices and testing conditions. Together, these features constitute meaningful methodological and conceptual advancements that refine and extend prior findings.

We hypothesized that higher total CTQ scores (indicating more or more severe ACE) would be associated with reduced performance in the gradCPT. Furthermore, emotional ACE (abuse and neglect) were expected to demonstrate stronger negative associations with sustained attention compared to their physical counterpart. This prediction was grounded in neurobiological evidence indicating that emotional abuse and neglect are particularly linked to alterations in prefrontal and fronto-limbic circuits that underlie attentional control and executive functioning [1, 15, 21]. In contrast, physical ACE have been associated with more heterogeneous and less domain-specific white matter changes. Consequently, we predicted that emotional ACEs would show stronger detrimental effects on sustained attention performance.

## Methods

### Participants

**Fig. 1:**
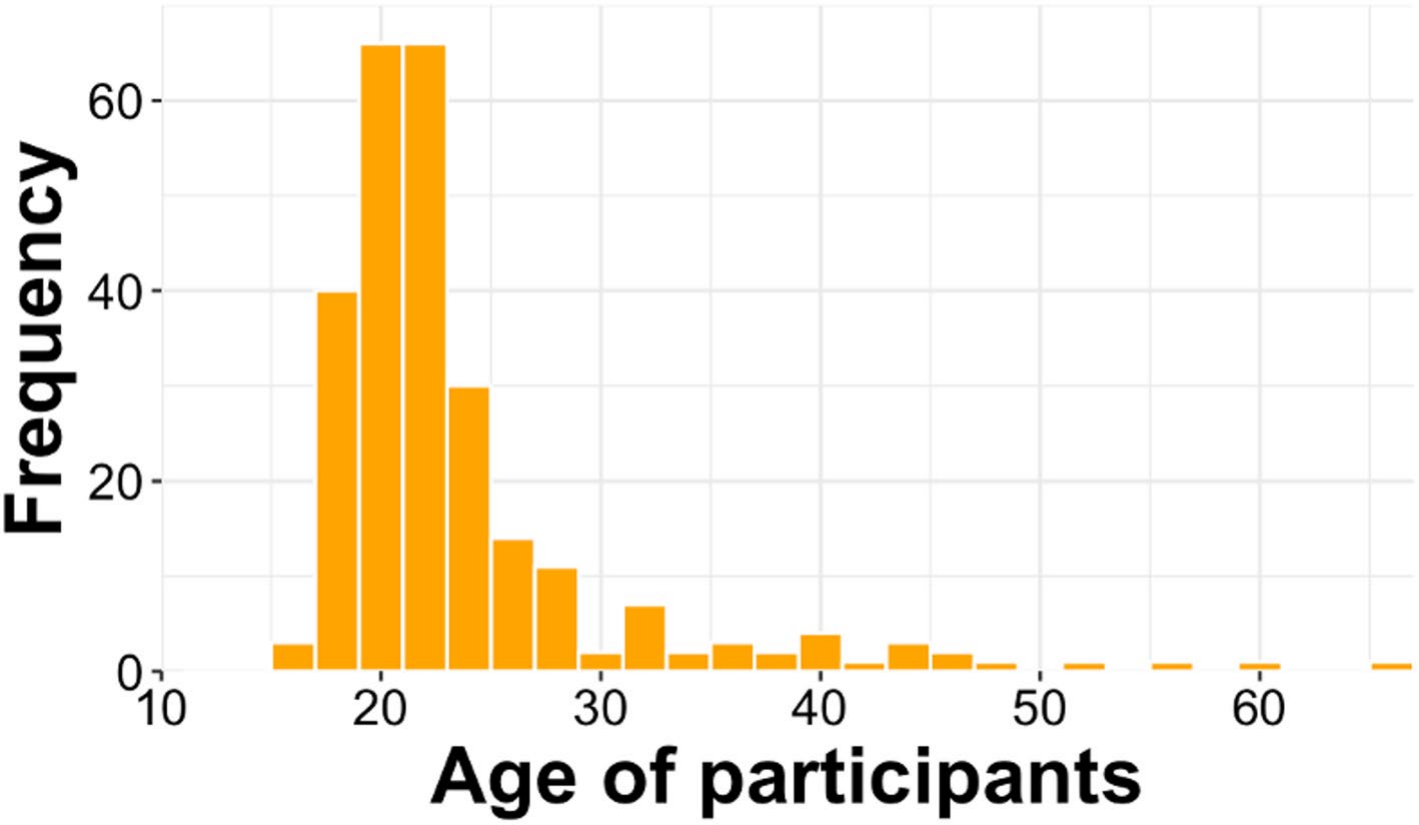
Histogram of Participants age

262 subjects between 18 and 66 (*M* = 24.29; *SD* = 7.7, see Fig. 1) were recruited via the local university online platform, social networks, public posters and flyers. Inclusion criteria were legal age (> 18 years) and access to an Internet-enabled device (e.g. smartphone, tablet, PC) with stable Internet connection. 72.5% self-reported the female gender, 25.6% reported male and 1.9% stated the diverse gender option. 80.9% of subjects reported no current diagnosis of a psychological or neurological disorder. As a result of the online nature of this study, no data about specific diagnoses was collected. The psychological and neurological health status was assessed dichotomously to function as a predictor in our analysis. 70% of subjects conducted the study on a handheld device with a touchscreen (i.e., tablet or smartphone) while the remaining 30% used a PC.

### Measures

ACE were assessed using the German version of the Childhood Trauma Questionnaire (CTQ; [12]. This 28-item self-report instrument includes five subscales: emotional abuse, physical abuse, sexual abuse, emotional neglect, and physical neglect. For each of the five subscales, five items were rated on a 5-point Likert scale ranging from 1 to 5, with higher scores indicating greater trauma exposure. Three additional items formed a trivialization scale to detect underreporting. These were not included in the total and sub scores. Reverse-coded items were recoded prior to analysis. A total CTQ score was computed by summing all 25 items. In addition, emotional and physical subscale scores were computed by summing the respective abuse and neglect subscales. In the analysis, the sexual abuse scale of the CTQ was excluded. This subscale measures both physical and emotional aspects of ACE and therefore makes it difficult to distinguish the different aspects of these dimensions.

**Fig. 2:**
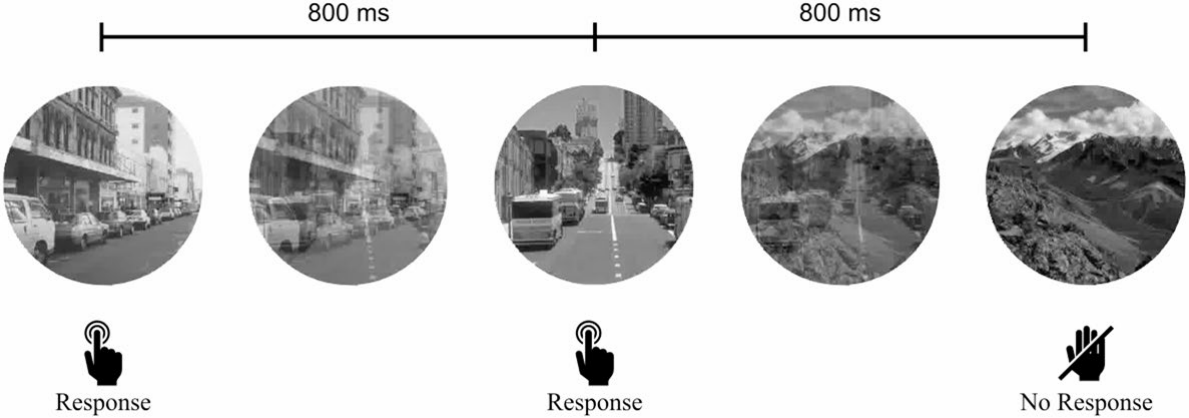
The gradual onset Continuous Performance Task was used to assess sustained attention parameters. Subjects viewed a continuous stream of complex scenes, constantly morphing into each other. 90% city scenes served as go trials and 10% mountain scenes served as nogo trials. Transitions between scenes lasted 800 ms

Sustained attention was assessed using the gradual onset gradCPT [2]. In contrast to other go/no-go tasks, stimuli in the gradCPT transition constantly into each other without abrupt on- and offsets. This allows for the continuous assessment of attentional states. With gradual transitions between visual stimuli, small fluctuations in sustained attention can also be captured, as these are not masked by cues, e.g. small breaks between the stimuli. This distinct feature helps reducing external cueing and therefore attracting endogenous attentional control [2, 4, 19]. Participants viewed a sequence of 300 grayscale images, with 89.3% depicting city scenes (go stimuli) and 10.7% mountain scenes (no-go stimuli), with each transition lasting 800 ms (see Fig. 2). Participants were instructed to respond to city scenes (i.e. press the space bar on their PC or tap the screen on their handheld device) and withhold responses to mountain scenes. The task lasted four minutes and was preceded by a short practice phase with feedback. Discrimination ability (d’) and decision criterion were calculated using standard signal detection theory [20]. Reaction times (RTs) for correct go trials were computed according to Esterman et al. [2]. From these, the mean RT and RT Coefficient of Variation (RTCoV,standard deviation divided by the mean) were computed.

### Procedure

Data collection was conducted entirely online. The technical implementation of the study was carried out using the platforms Research Electronic Data Capture (REDCap) version 14.0.1; [10], GitHub Pages (version 3.15.0), the web application service EmailJS and the development environment Visual Studio Code (version 1.96.0). After providing informed consent and reading a disclaimer regarding the potentially distressing content of the CTQ, subjects generated an anonymized subject code, that was later used to match CTQ and gradCPT data and completed a brief demographic questionnaire. Afterwards, they answered the CTQ. Subsequently, they were automatically directed to the gradCPT, which was implemented via GitHub Pages (version 3.15.0) using a script provided by www.TestMyBrain.org. Survey data were collected using REDCap, and task data were transmitted to local university servers using EmailJS. A custom Python script (version 3.9.6, [23] was used to match questionnaire and task data via the anonymized subject codes in Visual Studio Code (version 1.96.0). The study was approved by the local ethics committee of the University of Magdeburg, and all participants provided written informed consent in accordance with the Declaration of Helsinki.

### Analyses

Statistical analyses were performed using R [18] and RStudio version 2024.12.0 + 467 [17] and followed the approach by Vogel et al. [24]. To assess ACE effects on multiple parameters of sustained attention, two multivariate multiple regression models were fitted using the standardized scores for d’, the decision criterion, mean RT and RTCoV as dependent variables. In the first model, primary predictor of interest was the total CTQ sum score. In the second model, sum scores for emotional and physical subscales as well their interaction served as primary predictors. Covariates in both models included self-reported gender and mental health, education level, linear and quadratic effects of age as well as hardware used (e.g. touchscreen or computer). Dependent variables as well as predictors were z-scored prior to the analysis to assure comparability. Multicollinearity between predictors was assessed using the generalized variance inflation factor (GVIF) [6]. We report the adjusted GVIF, which takes predictors with more than one degree of freedom into account. Values below 4 are generally considered unproblematic, whereas values above 5 to 10 may indicate substantial multicollinearity [16]. Standardized regression coefficients are reported including the 95% confidence intervals. Model assumptions were assessed using graphical residual diagnostics. Histogram and QQ-Plots were used to assess normality and residuals vs. fitted values as well as standardized residuals were used to assess homoscedasticity and potential outliers. False discovery rate correction using the Benjamini–Hochberg procedure was applied to account for multiple comparisons.

## Results

### Descriptive statistics

**Fig. 3:**
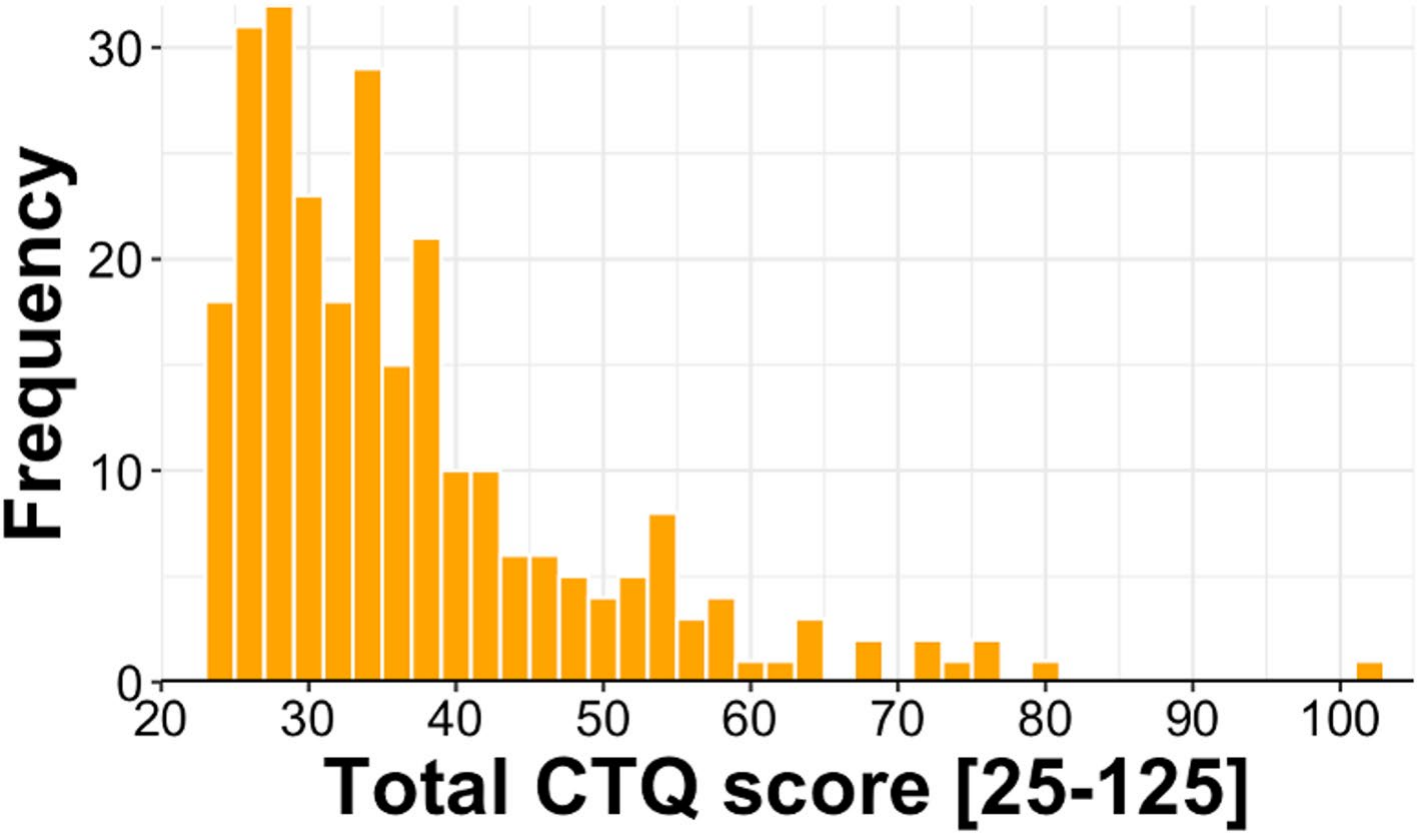
Histogram of Childhood Trauma Questionnaire Total Score distribution

CTQ Total Scores ranged from 25 to 103 (M = 37.25, SD = 12.1; median = 34; see Fig. 3), indicating considerable variability in childhood trauma exposure across participants. There was no evidence for underreporting in the sample, as no subject scored positive in the associated trivialization scale.

Descriptive analyses of the CTQ subscales revealed notable differences in score distributions. Emotional abuse (M = 9.44, SD = 4.52) and emotional neglect (M = 9.71, SD = 4.26) showed the highest means and greatest variability, indicating a broader spread of responses across the sample. In contrast, physical abuse (M = 5.69, SD = 1.73) and physical neglect (M = 6.67, SD = 2.32) were more narrowly distributed and clustered around the lower bound of the scale (min = 5 for all subscales), suggesting potential floor effects. Similarly, scores for sexual abuse (M = 5.74, SD = 2.34) were also low on average with limited variance. These patterns may reflect genuine differences in prevalence within the sample but also imply reduced statistical sensitivity for detecting effects in these subscales due to restricted variance.

Descriptive parameters of the gradCPT in our sample resembled those reported in a previous study, which also administered the task online [5]. On average, participants showed a d’ of 3.0 (SD = 0.75), a RT of 736 ms (SD = 76), a RTCoV of 0.19 (SD = 0.05), and a decision criterion of 0.71 (SD = 0.40).

### Inferential statistics

**Fig. 4:**
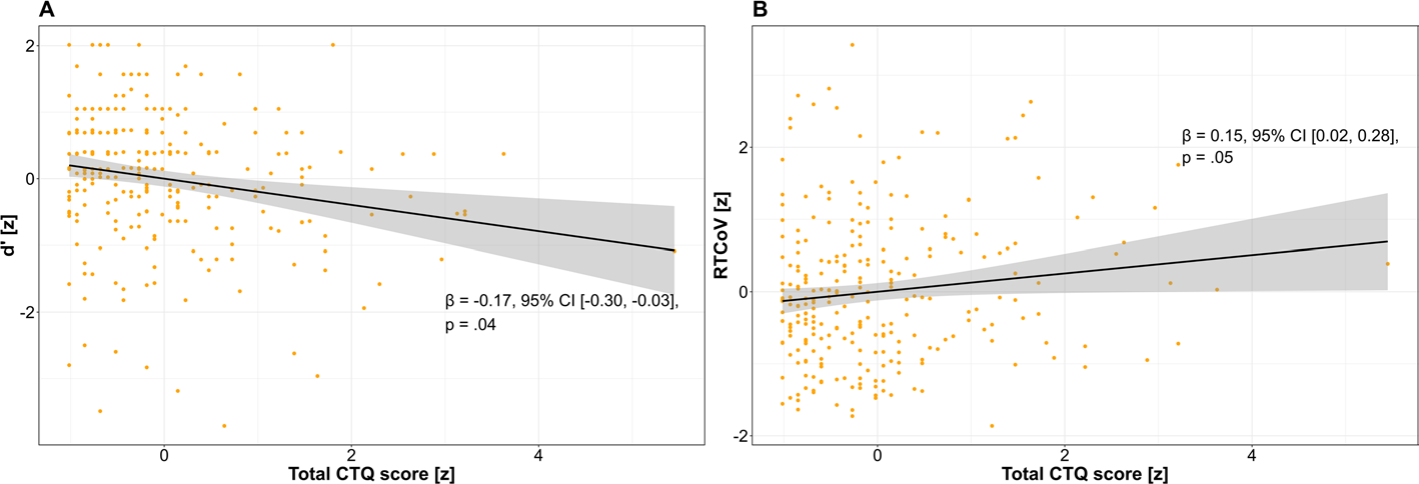
Significant effects of CTQ Total Score on sustained attention parameters. A: d’ was negatively associated with the CTQ total score indicating lower discriminatory ability with increasing ACE. B: Reaction time variability (RTCov) was positively associated with CTQ total score indicating less stable response performance with increasing ACE. Shaded area around regression lines indicates the standard error of the mean

The CTQ Total Score significantly predicted two key measures of sustained attention: Higher trauma exposure was negatively associated with the discrimination parameter *d’* (*b* = –0.17, 95% CI [–0.3, –0.03], *p* = 0.04, see Fig. 4a). For this prediction the model predicted 5% of variance (adjusted R^2^ = 0.05). In addition, higher CTQ total scores were positively associated with RTCoV (*b* = 0.15, 95% CI [0.02, 0.28], *p* = 0.05, see Fig. 4b), indicating less stable attentional performance over time. Here, the model explained roughly 7% of variance (adjusted R^2^ = 0.067). In addition, two demographic covariates showed isolated significant associations. Participants using touchscreen devices exhibited slower mean reaction times compared to those using a computer (*b* = −0.37, 95% CI [−0.12, −0.62], *p* = 0.02). We further observed quadratic effects of age RT (*b* = 1.2, 95% CI [0.53, 1.87], *p* < 0.001). For this model, graphical inspection of the residual distribution and QQ-plots indicated that residuals were approximately normally distributed. All predictors showed acceptable GVIF values (< 1.1), except for age (6.02) and age^2^ (6.05), which were strongly correlated (r = 0.97). This is expected as age^2^ was derived from age and does not pose a concern for model stability or interpretation. No outliers were removed.

The second model included combined scores for emotional and physical ACE (abuse and neglect) as well as their interaction. This analysis revealed no significant effect of emotional or physical ACE on sustained attention performance after correction for multiple comparisons. Uncorrected p-values only indicated a marginally significant effect of emotional ACE on the response criterion (*b* = 0.16, 95% CI [0, 0.33], *p* = 0.05) with roughly 8% explained variance (adjusted R^2^ = 0.077). However, as the 95% confidence interval contains zero, this effect needs to be interpreted with care as it suggests that a true effect could be absent or very small. No other significant main or interaction effects were observed (all *p* > *0.1*5), underscoring the relevance of cumulative trauma load (CTQ Total Score) over domain-specific effects (emotional vs. physical) in predicting sustained attention outcomes. For this second model, residual distribution and QQ-plots of the residuals indicated again normal distribution of residuals. GVIF values were again acceptable (< 1.4) except for age and age^2^ as in the previous model. Again, no outliers were removed.

Importantly, in neither of the models was the presence of a neurological or psychiatric disease a significant predictor of sustained attention performance (*p* = 0.37).

## Discussion

The present study confirms and extends previous findings on the negative association between ACE and cognitive performance in adulthood. Specifically, we found that higher cumulative childhood trauma, as assessed by the CTQ, was significantly associated with reduced d′ and increased RTCoV in the gradCPT. These results suggest that a higher overall burden of childhood trauma may impair sustained attentional abilities later in life, a finding with important implications for both clinical practice and preventive interventions.

Our study thus confirms and refines previous data [24]. In this study, ACE were operationalized through a general adversity score derived from household dysfunction variables (e.g., parental substance abuse, divorce). In contrast, the present study employed a more differentiated assessment using the CTQ, which distinguishes between emotional, physical, and sexual abuse, as well as emotional and physical neglect. Despite these methodological differences, our results robustly corroborate the notion that ACE may be associated to sustained attention deficits, underscoring the replicability and generalizability of this association across instruments and samples. In fact, showing that different methodological approaches yield convergent results provides strong evidence for the robustness and validity of this association. Such convergent evidence minimizes method-specific biases, enhances both internal and external validity, and strengthens the theoretical interpretability of the results. Some variation in effect size and pattern across studies may stem from differences in how childhood adversity is conceptualized. The CTQ focuses specifically on experiences of abuse and neglect, whereas broader adversity indices, such as those used by Vogel et al. [24], also include contextual factors like household instability or parental conflict. These broader measures capture environmental stressors that may affect attentional control through mechanisms such as chronic stress or socioeconomic disadvantage, while the CTQ targets more direct trauma-related pathways. Acknowledging these conceptual differences helps explain minor discrepancies between studies and highlights that adversity-related cognitive risks likely arise from multiple, partly overlapping mechanisms. Notably, the emotional and physical subscale scores of the CTQ did not show significant individual or interaction effects in predicting attentional outcomes. This finding contrasts with our hypothesis, which was based on prior neurobiological research suggesting that emotional abuse and neglect are particularly associated with alterations in prefrontal and fronto-limbic circuits implicated in attentional control. Several methodological factors may have contributed to the absence of significant subscale effects. In particular, the restricted variability and relatively low endorsement of physical maltreatment items likely reduced statistical sensitivity. For the emotional subscale, a marginal effect was only observed before correcting for multiple comparisons. However, as the 95% CI included 0, this result is not conclusive. A true effect could be absent or have low effect sizes. Future studies with higher statistical power could provide a clearer understanding of these association.

Furthermore, the use of the gradCPT in an entirely online context proved to be a reliable and easy approach for capturing subtle fluctuations in sustained attention. The fact that robust effects were observed despite variation in testing conditions and device types (e.g., touchscreen vs. computer) supports the ecological validity and robustness of the gradCPT as a tool for large-scale cognitive assessment.

However, several limitations should be considered when interpreting these findings. The cross-sectional design of the study does not allow for causal conclusions regarding the directionality of the observed effects. Since childhood trauma was assessed retrospectively via self-report, the results may be influenced by memory biases or underreporting—especially in sensitive areas such as physical abuse. Although the CTQ includes a trivialization scale to account for such tendencies, it cannot fully eliminate these concerns. In addition, the online recruitment strategy entails potential self-selection bias; individuals who voluntarily participate in online cognitive testing may differ systematically in motivation, education, or psychological traits, which limits the generalizability of the findings. Also, unmeasured confounding factors such as current medication use, sleep quality, or substance consumption might have influenced attentional performance. Although aggregating CTQ subscales into a total score increases reliability and statistical power, it also masks potentially distinct mechanisms linked to specific adversity types.

Another issue relates to the variance and distribution of specific trauma subtypes. In particular, physical abuse was reported relatively infrequently in this sample, which may have limited the ability to detect subtype-specific effects. This could partially explain the absence of significant findings for emotional and physical CTQ subscales. Furthermore, our approach of combining the CTQ subscales for the specific analysis of emotional and physical ACEs may have reduced granularity and obscured potential differences in the effects of the individual subscales. In addition, while the online implementation of the gradCPT proved generally reliable, device-related differences, such as slower response times on touchscreens, might have introduced minor variability in performance measures. Lastly, the sample was relatively homogeneous in terms of age, education, and gender, which may restrict the generalizability of the findings to more diverse populations with higher or more varied trauma exposure. In addition, there are other limitations for the generalizability of the results. The online recruitment strategy entails potential self-selection bias: individuals who voluntarily participate in online cognitive testing may differ systematically in motivation, education, or psychological traits. Also, unmeasured confounding factors such as current medication use, sleep quality, or substance consumption might have influenced attentional performance. Although aggregating CTQ subscales into a total score increases reliability and statistical power, it also masks potentially distinct mechanisms linked to specific adversity types. Due to the great age range of the participants, recall bias could be a potential limitation of this study. With subjects up to 66 years old, memories of childhood trauma could be less accurate. Nevertheless, as the majority of participants (78.2%) were aged between 18 and 25, the overall impact of recall bias is likely limited. Overall, the sample is relatively young, predominantly female, and mostly recruited via a university-related platform. These attributes are important to mention because the presence of ACE is e.g. related to a person's social status. Low social status is a predictor of higher scores on the emotional and physical subscales. Also, there are gender-specific differences on the sexual abuse subscale: women report this ACE dimension significantly more often than men [27]. Due to the selectivity of the sample, the generalizability of the results to the overall population is limited.

## Conclusion

The current study provides further evidence for the growing body of research suggesting that ACE could have lasting negative effects on cognitive functioning, specifically on sustained attention in adulthood. By using the CTQ to capture cumulative trauma exposure and the gradCPT to assess attentional control, we observed that higher childhood trauma is significantly associated with decreased discrimination ability and increased response time variability. While the observed relationships between cumulative trauma load and attentional measures are consistent with previous work, the cross-sectional and retrospective nature of this study does not allow for causal interpretations. Methodological factors such as recall bias in the adult sample and restricted variance in specific trauma subtypes should be considered when interpreting the results. However, our data demonstrate that assessing cumulative ACE exposure using the CTQ and measuring sustained attention through an online version of the gradCPT can yield meaningful insights into the cognitive correlates of early adversity. Although no significant effects of specific trauma subtypes were observed, the cumulative trauma load emerged as a reliable predictor of attentional outcomes, suggesting that the overall burden may be more impactful than the individual type of trauma. Future studies should employ longitudinal designs and explore potential moderating and mediating variables. Of interest would be resilience, mental health status, and neurobiological factors to better understand the mechanisms linking early adversity to adult cognitive functioning. For instance, resilience factors might buffer the impact of ACE on attentional/cognitive performance [8]. Conversely, psychiatric symptoms like depression or PTSD could mediate or amplify this association [11]. These insights may contribute to the development of more targeted prevention and intervention strategies for individuals with a history of childhood trauma.

## Data Availability

The data supporting the findings of this study are available in the Open Science Framework (OSF) repository at 10.17605/OSF.IO/KU2WT, licensed under CC BY 4.0.
